# Genetic parameters and inbreeding effects for production traits of Thai native chickens

**DOI:** 10.5713/ajas.18.0690

**Published:** 2019-01-02

**Authors:** Siriporn Tongsiri, Gilbert M. Jeyaruban, Susanne Hermesch, Julius H. J. van der Werf, Li Li, Theerachai Chormai

**Affiliations:** 1 Animal Genetics and Breeding Unit, University of New England, Armidale, NSW 2351, Australia; 2 Kabinburi Livestock Research and Breeding Center, Bureau of Animal Husbandry and Genetic Improvement, Department of Livestock Development, Prachin Buri 25110, Thailand; 3 School of Environmental and Rural Science, University of New England, Armidale, NSW 2351, Australia

**Keywords:** Indigenous Chicken, Heritability, Genetic Correlation, Inbreeding Depression, Tropical Climate

## Abstract

**Objective:**

Estimate genetic parameters, the rate of inbreeding, and the effect of inbreeding on growth and egg production traits of a Thai native chicken breed Lueng Hang Kao Kabinburi housed under intensive management under a tropical climate.

**Methods:**

Genetic parameters were estimated for weight measured at four weekly intervals from body weight at day 1 (BW1D) to body weight at 24 weeks (BW24) of age, as well as weight at first egg, age at first egg (AFE), egg weight at first egg, and total number of eggs (EN) produced during the first 17 weeks of lay using restricted maximum likelihood. Inbreeding depression was estimated using a linear regression of individual phenotype on inbreeding coefficient.

**Results:**

Direct additive genetic effect was significant for all traits. Maternal genetic effect and permanent environmental hen effects were significant for all early growth traits, expect for BW24. For BW24, maternal genetic effect was also significant. Permanent environmental hen effect was significant for AFE. Direct heritabilities ranged from 0.10 to 0.47 for growth traits and ranged from 0.15 to 0.16 for egg production traits. Early growth traits had high genetic correlations between them. The EN was lowly negatively correlated with other traits. The average rate of inbreeding for the population was 0.09% per year. Overall, the inbreeding had no effect on body weight traits, except for BW1D. An increase in inbreeding coefficient by 1% reduced BWID by 0.09 g (0.29% of the mean).

**Conclusion:**

Improvement in body weight gain can be achieved by selecting for early growth traits. Selection for higher body weight traits is expected to increase the weight of first egg. Due to low but unfavorable correlations with body weight traits, selection on EN needs to be combined with other traits via multi-trait index selection to improve body weight and EN simultaneously.

## INTRODUCTION

The poultry industry provides an important protein source and household income to many smallholders in Thailand. The Thai native chicken (TC) is the most popular local breed among rural farmers due to its relative superiority with respect to disease resistance and adaptation to harsh environmental conditions compared to non-local breeds. Also, it is considered an important economic resource for rural people. Its importance to the Thai economy was clearly exposed during the avian influenza outbreak in 2004 when the Thai poultry industry could not import or export poultry products and the industry relied entirely on TC.

There are 12 breeds of TC in Thailand. The TC is small in size, ranging in weight from 1 to 1.5 kg by 2.7 months of age, and also has low egg production, with 30 to 40 eggs (20 to 24 chicks) per hen per year, which is well below the annual household requirement. Yenjai and Sinnak [1] estimated that about 40 chicks (equivalent to 12 to 20 marketable birds) per hen per year are needed to support a family of four. Therefore, all growth, egg and chick producing ability traits of the TC need to be improved for sustainable poultry production in rural Thailand.

In 2003, the Department of Livestock Development (DLD) in Thailand established a breed-improvement program for a popular TC breed known as Lueng Hang Kao Kabinburi (LHKK) chicken to improve its productivity. The main objective of this improvement program was to maintain the breed characteristics. Several years of within-line selection and random mating resulted in improved uniformity of breed characteristics. However, the improvement in performance in relation to growth and egg production was limited and requires further attention.

Establishment of a selection program for improvement in growth, egg and chick production requires the estimation of genetic parameters for these traits. Using within-line selection on a small poultry population is expected to increase the rate of inbreeding in the population, and the increase in rate of inbreeding may lead to undesirable effects such as a decline in performance and loss of vigor [2]. Therefore, estimation of the rate of inbreeding is required and its influence on production traits needs to be explored before the application of any selection scheme. Studies on genetic parameters and inbreeding effects in native chickens are limited, especially for TC. The objective of this study was to estimate genetic parameters, the rate of inbreeding, and the effect of inbreeding on growth and egg production traits of TC housed in intensive management under a tropical climate.

## MATERIALS AND METHODS

### Breeding program for Lueng Hang Kao Kabinburi chickens

Growth and egg production data used in this study were from the native breed improvement program. The native poultry breed improvement program was initiated in 2003 at the Kabinburi Livestock Research and Breeding Center, DLD in Thailand. The Livestock Research Centre is located in the Eastern region of Thailand and it is situated between 14.0478° North latitude and 101.3725° East longitude. It receives an average annual rainfall of about 1,532 mm and average daily temperature varied from 15°C to 40°C within the five years recorded from 2003 to 2007. The initial parent stock of 70 cocks and 350 hens were sourced across the country as the founding gene pool of LHKK chickens. Each cock was mated to five hens by artificial insemination. Fertile eggs were collected for two weeks and hatched in the incubator as the same batch. Each hen produced about 60 day-old chicks per year. Annually, 70 males and 350 females were selected as replacement birds of each generation. Birds kept for dual purposes and they were selected based on breed characteristics, including the type of comb, face color, eyes, beak, skin, tail, shank, body plumage, neck, and saddle plumage.

During the first 3 weeks after hatching, all chicks were housed in deep-litter housing with an average of 7.5 chicks per square meter (m^2^) and 24 hours of light schedule. They were fed *ad libitum* with a ration containing 190 g of crude protein (CP) and 11.7 MJ metabolizable energy per kg. For the next 18 weeks, chicks were moved to grower pens and were allowed to scavenge outside during daytime and were sheltered inside at nighttime, at a rate of 8 chickens per one square meter (m^2^). The light schedule was 12 hours per day and feeding was *ad libitum* with a ration containing 150 g of CP and 11.9 MJ metabolizable energy per kg. At 22 weeks of age, 70 cocks and 350 hens were selected as replacements and moved to individual battery cages. The light schedule was steadily increased by 1 hour per week until it reached 16 hours per day. Some fresh grass was fed as supplementation during starter and grower periods. During the laying period, they were fed with layer feed at a daily rate of 120 g for hens and 150 g for cocks (from 22 weeks of age until culling) with 160 g CP and 12.1 MJ metabolizable energy per kg. Birds were vaccinated for Marek’s disease, New Castle disease, Infectious Bronchitis disease and Fowl Pox disease as recommended by DLD.

### Traits studied

Traits studied were weight measured at four weekly intervals from hatch to 24 weeks of age, weight at first egg, age at first egg, egg weight at first egg (EWFE) and total number of eggs produced during the first 17 weeks of lay. The birth weight at hatch was defined as the individual body weight at day 1 (BW1D, in grams) of the chick when measured on the first day of age and is the starting point for recording growth performances. Subsequently, individual BW of chicken were measured at 4 (BW4), 8 (BW8), 12 (BW12), 16 (BW16), 20 (BW20), and 24 (BW24) weeks of age. The BWs were measured in grams at 4 and 8 weeks of age and in kilograms thereafter. The BW at first egg (BWFE) is the BW (in kilograms) of the hen when the first egg laid while hens were in the individual battery cage. Age at first egg (AFE) is the age of the hen (in days) when her first egg was laid. The EWFE is the weight (in grams) of the first egg of an individual hen. Number of eggs (EN) is the total number of eggs per hen from the start of lay until the end of the 17th week after the start of lay.

### Data preparation

Phenotypic records within three standard deviations from the population mean were kept for this analysis. Main fixed effects identified were year, hatch within year and sex of bird. There were five years of hatch (from 2003 to 2007) with an average of 17 hatches per year. The BW traits were recorded when the LHKK project started and the egg production traits of LHKK were recorded a year later. Thus, all BW traits were recorded for five generations, only four generations of data were recorded for AFE and EWFE, and three generations of data for EN. Trait observations without hen identification were removed, which accounted for 1% to 6% of the observations. Parent cocks and hens were known for each bird, and five generations of pedigree were available for all birds with records. The total number of individual birds in the pedigree was 17,883 and the number of birds with records was 11,588, and they descended from 486 cocks and 1,461 hens. The same pedigree was used for all traits in estimating genetic parameters.

### Statistical analyses

The PROC Mixed procedure in SAS program [3] was used to calculate descriptive statistics and to identify significant fixed effects for each trait in a model with the cock effect fitted as random. Year and hatch within year were significant for all traits, and sex was significant for all growth traits. Significant fixed effects were included in the model to estimate genetic parameters.

Genetic parameters were estimated using restricted maxi mum likelihood methods by fitting an animal model in the WOMBAT software package [4]. The influence of maternal genetic effect and permanent environmental hen effects were explored by comparing four different models for each trait. Those four models were as follows,

$$\begin{array}{l} \text{Model A:}\;Y=\mathrm{Xb}+Z_{1}a+e\\ \text{Model B:}\;Y=\mathrm{Xb}+Z_{1}a+Z_{2}m+e\\ \text{Model C:}\;Y=\mathrm{Xb}+Z_{1}a+Z_{3}\mathrm{pe}+e\\ \text{Model D:}\;Y=\mathrm{Xb}+Z_{1}a+Z_{2}m+Z_{3}\mathrm{pe}+e \end{array}$$

Where: **Y** = observation’s vector of the trait; **b** = vector of fixed effects (year, hatch within year, and sex effects); **a**, **m**, and **pe** are the vectors of direct additive genetic effect, maternal genetic effect and permanent environmental hen effects, respectively; **e** = vector of random residual effect; **X**, **Z_1_**, **Z_2_**, and **Z_3_** are incidence matrices relating records to the fixed, direct additive genetic effect, maternal genetic effect and permanent environmental hen effects, respectively.

The variance components for the random effects were de noted as

$$\text{var }(\text{a})=A{\sigma^{2}}_{\text{a}},\text{var }(\text{m})=A{\sigma^{2}}_{\text{m}},\text{var }(\text{pe})=I{\sigma^{2}}_{\text{pe}}$$

where **A** is a numerator relationship matrix. The covariance between direct additive genetic effect and maternal genetic effect was assumed to be zero.

The log likelihood ratio test was used to evaluate the sig nificance of fitting various random effects in the models. A chi square distribution with one degree of freedom was used as the critical test statistic. The inclusion of the effect was considered significant when twice the difference in the Log likelihood of nested models differing by one random factor was greater than the critical value of 3.84 (α = 0.05). Significant random effects (p<0.05) were included in the final model. Estimated heritabilities and genetic correlations were based on the best model identified in the previous step. Estimated genetic parameters and the log likelihood for each model are given in Supplementary Table S1 and S2. The heritabilities and correlations for all traits were estimated using univariate and bivariate analysis, respectively. Due to an inadequate number of observations for EN, only model A was used to estimate the genetic parameters for this trait. Genetic trends for each trait was predicted by regressing the EBVs for each trait on the birth year of the animal.

WOMBAT was also used to estimate inbreeding coefficients [4] based on the algorithm proposed by Tier [5] for implementation of the tabular method. A linear regression of individual phenotype on inbreeding coefficient was used to estimate inbreeding depression using the PROC Mixed procedure of the SAS program. All fixed effects were included in the model with inbreeding coefficient as a covariate and cock as a random effect.

## RESULTS

### Data statistics

Average BW of LHKK chickens increased with age, ranging from 31 g for BW1D to 2.12 kg at BW24 (Table 1). They reached AFE at around 28 weeks of age, with an average BWFE of 2.05 kg. The average EWFE was 37 g, and the average EN was 54 eggs per hen. The number of observations decreased from BW1D to BW24 by 47% due to the culling of birds for non-breed characteristics at different ages.

### Genetic parameters

#### Variance components

Model D, including direct additive genetic, maternal genetic effect and permanent environmental hen effects was the most appropriate (p<0.05) for BW1D, BW4, BW8, BW12, BW16, and BW20. Model B, including direct additive genetic and maternal genetic effect was most appropriate for BW24, and model C, including direct additive genetic effect and permanent environmental hen effects was the best model for AFE. Model A, including direct additive genetic effect only was found best for BWFE and EWFE. Table 2 summarizes the variance components estimated from these models.

#### Heritability (h^2^)

The heritabilities were generally low to high and standard errors were low (Table 2). Direct heritabilities ranged from 0.10 to 0.47 across traits and standard error ranged from 0.02 to 0.06 across most traits, except for EN, which had a higher standard error of 0.11. The lowest and highest heritability were obtained on BW1D (0.10) and BWFE (0.47), respectively. The heritability of growth traits gradually increased as the birds grew older. For egg production traits, the heritabilities were consistently low. Heritabilities for maternal genetic effects were low to moderate for all growth traits, except for BWFE, and ranged from 0.02 (0.02) to 0.26 (0.04). Maternal heritabilities declined when age of bird increased during early growth traits (BW1D to BW20). The proportions of phenotypic variance explained by permanent environmental hen effects were low to moderate for all growth traits and AFE, except for BW24 and BWFE, and ranged from 0.04 (0.01) to 0.27 (0.03).

#### Genetic correlations (r_g_)

Genetic correlations between growth traits from BW4 to BW24 were all positive (Table 3). Genetic correlations estimated between growths traits measured after four weeks of age were high. The correlations between BW1D and BWFE had lower correlations with other growth traits. Correlations between growth traits and AFE, and EN with other traits were lower (Table 3). The BWFE had a positive correlation with all traits, except with EN, which was negatively correlated with BWFE.

Genetic correlations between AFE and other traits were variable. The genetic correlations between AFE and growth traits were positive, except for BW4, BW8, and BW12. High genetic correlations were estimated between AFE and BW1D (0.63) and BWFE (0.72). Genetic correlations between EWFE and growth traits were high positive and ranged from 0.37 to 0.71. EN was negatively correlated with all traits and the correlations ranged from −0.10 to −0.73. Negative genetic correlations between growth traits and EN indicate that selection for higher growth rate would have reduced the number of eggs laid. Estimated correlations between maternal genetic effects were high between growth traits and ranged from 0.65 to 1.00 (Table 3).

#### Permanent environmental hen effects and phenotypic correlations (r_p_)

The permanent environmental hen effect had positive correlations for growth traits, and ranged from 0.57 to 0.99 (Table 4). Low negative correlations of permanent environmental hen effects were observed between BW1D and other growth traits (−0.17 to −0.13). The correlations between AFE and growth traits were positive (0.06 to 0.38), except between AFE and BW1D (−0.05).

Overall, phenotypic correlations were positive between growth traits (Table 4). The BW1D had low correlations with other growth traits which ranged from 0.12 to 0.22. Phenotypic correlations between growths traits measured after four weeks of age were moderate to high and ranged from 0.29 to 0.90. Estimated phenotypic correlations of AFE and EN with other traits were low, and were not significantly different from zero.

### Rate of inbreeding

**Figure 1:**
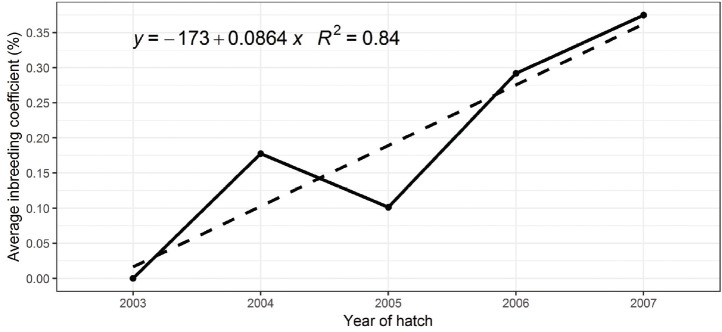
Change in mean inbreeding level for growth traits of Lueng Hang Kao Kabinburi chickens measured at four weekly intervals from day old (BW1D) to 24 weeks of age (BW4, BW8, BW12, BW16, BW20, and BW24) over five generations.

Figure 1 shows the level of inbreeding for the animals included in this study. The inbreeding coefficient increased across years at a rate of 0.09% per year. This level of inbreeding of 0.09% per generation is within the generally accepted level, which is often considered to be around 1% per generation. The number of males used in each generation as parents was relatively large (111 cocks per generation by average) and along with limited selection have contributed to the lower level of inbreeding in the population.

### Inbreeding coefficient and inbreeding effects

The number of inbred birds, average inbreeding level and change in trait value per 1% of inbreeding, across generations, varied for traits (Table 5). Egg production traits were recorded on fewer than 2,000 hens over a period of less than three years. Therefore, they were not included in the calculation of inbreeding effects on trait means. For growth traits (BW1D to BW24), the proportion of inbred birds ranged from 6.33% to 8.07% and the average inbreeding coefficient of the population was 0.25%. The effect of 1% increase in inbreeding level on traits varied from −0.32 g to 0.004 kg and the inbreeding effect in % of trait mean varied from −0.29% to 0.27%. Generally, inbreeding has very little effect on BW of LHKK chicken. However, inbreeding had a low negative effect on BW1D (p<0.0001) by reducing it by 0.09 g (−0.29% of trait mean), and low positive effect on BW12 (p<0.05) by increasing it by 0.003 kg (0.27% of trait mean).

### Estimated genetic trends

**Figure 2:**
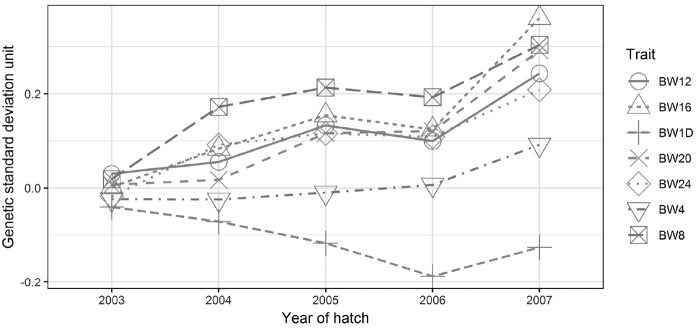
Genetic trend for five years, expressed in unit of genetic standard deviation (GSU), for growth traits of LHKK chickens measured at four weekly intervals from day old (BW1D) to 24 weeks of age (BW4, BW8, BW12, BW16, BW20, and BW24).

Genetic trends of growth traits expressed in units of genetic standard deviation unit (GSU) are shown over a five year period in Figure 2. Genetic trends increased for all growth traits, except for BW1D which showed a small negative genetic trend. For BW from 4 to 24 weeks of age, the genetic trends were positive. Genetic trends were −0.03±0.03, 0.03±0.02, 0.06±0.05, 0.05±0.04, 0.08±0.06, 0.07±0.05, and 0.05±0.04 GSU per year for BW1D, BW4, BW8, BW12, BW16, BW20, and BW24, respectively. Estimated standard errors indicated that the genetic means were was not significantly different from zero. The results indicated that the random mating breeding program and breed characteristic selection of LHKK chicken have no impacts on early growth trait (BW1D and BW4). Moreover, it has slightly positive impacts on late growth traits (BW16 and BW20).

## DISCUSSION

Estimated mean BWs of LHKK chickens were similar to average growth performance of four other indigenous chicken breeds managed under tropical Thailand conditions [6,7]. The LHKK chickens reached AFE earlier (10 days) and laid a smaller EWFE of about 2 g less than Pradu Hang Dam chickens [6]. Mean EN estimated for LHKK was very similar to the mean reported by Mekky et al [8] for crossbred chickens in Egypt.

Heritabilities of direct additive genetic effects were higher than maternal heritabilities and permanent environmental hen effects ratio for all growth traits, except for BW1D. This is because preovipositional maternal effects influence egg size and egg quality and thereby, influences chick weight at hatch [9]. Therefore, the BW1D has higher maternal influence. However, in this study, the chicks were reared without their hens, therefore, the maternal influence on other growth traits was low despite this carry over effect of the hen from BW1D. Heritabilities of direct additive genetic effect for growth traits were within the range published for native chickens. Na-Rungsri et al [10] estimated heritabilities of 0.43, 0.46, and 0.39 for BW8, BW12, and BW16 of Pradu Hang Dam, a native chicken in Thailand. Estimated heritabilities for egg production traits were in agreement with Boonkum et al [6] who also reported low heritabilities for AFE, EWFE and EN, and high heritability for BWFE of Pradu Hang Dam and Chee breed. Estimated heritabilities for egg production traits were also in agreement with the values reported for commercial egg laying chickens [11–14]. Moderate heritabilities for direct additive genetic effect of growth and egg production traits indicate that selection for higher growth or higher egg number could be applied to improve performances of LHKK chicken.

Genetic maternal and permanent environmental hen effects influence on growth and egg production traits were explored. Both maternal genetic effect and permanent environmental hen effects influenced all growth traits except for BW24. This is in agreement with the study by Ghorbani et al [15] on native chickens in Iran. Ghorbani et al [15] concluded that maternal genetic effects and permanent environmental hen effects should be included in an animal model for estimation of genetic parameters of growth traits of native chickens. Fitting direct additive genetic effect and permanent environmental hen effects gave the highest log likelihood estimates for AFE. Most of the studies fitted model with direct additive genetic effect to estimate parameter for AFE. Nevertheless, estimated parameters for AFE were within the range of estimates published by Sang et al [16], Dana et al [17], and El-Labban et al [18]. Models identified for BWFE, EWFE, and EN were also in agreement with Ghorbani et al [15] and Prado-Gonzalez et al [9] who suggested that BWFE, EWFE, and EN were not influenced by maternal genetic effect or permanent environmental hen effects, and the model with direct additive genetic effects only was the most suitable model for egg production traits.

The genetic correlations between growth traits were moder ate to high and in agreement with Dana et al [17] and Lwelamira et al [19] studies. They found high positive genetic correlations between growth traits, measured at 8, 12, 16, and 20 weeks of age for Horro chicken in Ethiopia and ecotypes chickens in Tanzania. Niknafs et al [20] also reported positive genetic correlations between BW at day 1, and 8 and 12 weeks of age (range from 0.37 to 0.91). High positive correlations between BW measured at early and later stages of growth suggest that selection for higher early growth would increase BW at maturity.

Total EN was negatively correlated with growth and re production traits. Lwelamira et al [19] and Niknafs et al [20] reported lower negative genetic correlations (−0.06 to −0.23) between EN and BW at different ages of indigenous chicken than the current study. Negative genetic correlations (−0.05 to −0.48) between EN and BWFE were also observed by Sang et al [16]. The estimated negative genetic correlation between EN and AFE in this study was similar to other studies. Negative correlations ranged from −0.15 to −0.81 between EN and AFE in the studies by Sang et al [16], Lwelamira et al [19], and Niknafs et al [21]. Aghazadeh Bokat et al [22] agreed that the sexual maturity was negatively correlated with egg production trait. For EN and EWFE, Sang et al [16] reported negative genetic correlations which ranged from −0.05 to −0.52 for Korean native chickens. However, Boonkum et al [6] reported no significant correlations (ranged from −0.02 to 0.02) between EN and EWFE for two TCs (Pradu Hang Dam and Chee). A low number of records for EN in this study resulted in high standard errors for the correlations between EN and other traits, and some of the correlations were not significantly different from zero. However, this study was aimed at quantifying the genetic potential of LHKK as a dual purpose chicken. Therefore, the EN was included in this analysis. The estimated negative correlation between EN and other growth and reproductive traits suggested that hens that reached sexual maturity earlier with a lighter BW would lay more eggs than hens that reach sexual maturity later with heavier BWs. However, the hens that reach sexual maturity later with heavier BW would lay heavier eggs.

Positive genetic correlations between BW at different ages and BWFE and EWFE from this study indicate that selection for higher growth would increase BWFE and EWFE. Niknafs et al [20] reported high positive genetic correlations of 0.41, 0.57, and 0.69 between BWFE and BW1D, BW8 and BW12, respectively. Moderate to high positive genetic correlations between BW measured at different ages and EWFE were in agreement with the study of Bahmanimehr [23]. Moreover, McNaughton et al [24] stated that chickens with heavy BW were hatched from heavy eggs tended to grow heavier and end up heavier at BWFE.

Improving BWFE would increase AFE and EWFE due to positive genetic correlations. Boonkum et al [6] and El-Labban et al [18] also found positive correlations between BWFE and AFE in native chickens in Thailand (Chee breed) (0.42), and Egypt (0.84), respectively. Moderate to high (0.36 to 0.78) genetic correlations between BWFE and EWFE were also reported by previous studies [6,16,18].

The AFE and EWFE had a high positive genetic correla tion of 0.66, which was in agreement with values reported for native chicken by El-Labban et al [18] and Sang et al [16], that ranged from 0.08 to 0.66. Koutoulis et al [25] stated that hens with longer AFE had heavier egg weight than hens with shorter AFE.

Estimated genetic correlations among the eleven traits of LHKK chickens suggest that the associations between growth and egg production traits are generally antagonistic. Chicken with heavier BW at maturity and heavier egg weight would increase household income. Selection for improving BW will lead to heavier individual egg weight of LHKK flock. However, it will have an unfavorable influence on the other egg production traits (AFE and EN). Selection for increased BW would result in increased egg weight through late sexual maturity and would lead to lower egg numbers. On the other hand, negative genetic correlations between EN and other traits indicate that hens with a lighter BW will reach sexual maturity earlier, and they can produce more eggs and chicks. Furthermore, lighter hens would lower feed cost due to lower consumption of feed. Therefore, a multi-trait selection strategy is required to achieve optimal growth and egg production in LHKK chickens.

Rate of inbreeding estimated in this study was not high enough to have any detrimental effect on performance or vigor of LHKK chickens. However, inbreeding of males and females had mixed influence on growth traits on LHKK chickens across 5 years. An increase of 1% of coefficient inbreeding decreased BW1D (−0.29 g). Niknafs et al [21] and Rahmanian et al [26] also reported a detrimental effect of higher inbreeding level on chick weight of native chicken. Chick weight reduced by −0.36 [21] and −0.11 g [26] per 1% increase in inbreeding in those studies.

Increase in inbreeding level also increased the BW measured after 4 weeks of age. Niknafs et al [21] reported improved BW at 8 and 12 weeks of age on Mazandaran native chicken in Iran by 9.32 and 2.30 g, respectively for a 1% increase in inbreeding level.

In conclusion, LHKK could be used as a dual purpose chickens. Moderate heritability estimated for growth and egg production traits suggested that both groups of traits could be improved through selection. However, antagonistic relationships shown in the phenotypic and genetic correlations between growth and some egg production traits, and effect of inbreeding rate suggested that both traits could not be improved simultaneously using additive selection. Therefore, multi-trait selection strategy using both groups of traits need to be implemented.

## SUPPLEMENTAL DATA



## Figures and Tables

**Table 1:** Description statistics of growth and egg production traits for LHKK chickens

Traits1)	No. of records	Mean	SD	Min	Max	CV (%)
BW1D (g)	11,588	30.93	3.38	22.00	40.00	10.93
BW4 (g)	11,201	218.91	56.68	46.00	379.00	25.89
BW8 (g)	10,807	642.08	138.74	260.00	1034.00	21.61
BW12 (kg)	9,777	1.10	0.21	0.52	1.67	19.09
BW16 (kg)	8,948	1.49	0.31	0.59	2.38	20.81
BW20 (kg)	7,643	1.81	0.41	0.64	3.00	22.65
BW24 (kg)	6,157	2.12	0.47	0.89	3.52	22.17
BWFE (kg)	1,428	2.05	0.25	1.40	2.75	12.20
AFE (d)	1,395	199.14	21.02	138.00	260.00	10.56
EWFE (g)	1,393	36.94	4.85	26.00	48.00	13.13
EN (eggs)	402	53.91	13.30	25.00	91.00	24.67

LHKK, Lueng Hang Kao Kabinburi; No. of records, number of records; SD, standard deviation; Min, minimum; Max, maximum; CV, coefficient of variation.

1) BW1D, body weight at day 1; BW4, BW8, BW12, BW16, BW20, BW24 are body weight at 4, 8, 12, 16, 20 and 24 weeks of age, respectively; BWFE, body weight at first egg; AFE, age at first egg; EWFE, egg weight at first egg; EN, total number of eggs laid from onset of lay to 17 weeks of lay.

**Table 2:** Estimated variance components of random effects of body weight and egg production traits of LHKK chickens

Trait1)	Model2)	σ^2^_a_	σ^2^_m_	σ^2^_pe_	σ^2^_p_	h^2^_a_	h^2^_m_	c^2^_pe_
BW1D (g)	D	1.20±0.23	2.96±0.51	3.13±0.38	11.51±0.33	0.10±0.02	0.26±0.04	0.27±0.03
BW4 (g)	D	232.65±32.31	47.98±19.11	91.40±17.03	1,141.78±20.34	0.20±0.03	0.04±0.02	0.08±0.02
BW8 (g)	D	2,645.90±264.98	301.01±130.00	379.14±104.73	7,860.71±153.07	0.34±0.03	0.04±0.02	0.05±0.01
BW12 (kg)	D	0.01±0.00	0.00±0.00	0.00±0.00	0.02±0.00	0.37±0.03	0.03±0.02	0.05±0.01
BW16 (kg)	D	0.01±0.00	0.00±0.00	0.00±0.00	0.04±0.00	0.30±0.03	0.04±0.02	0.04±0.01
BW20 (kg)	D	0.02±0.00	0.00±0.00	0.00±0.00	0.06±0.00	0.30±0.03	0.02±0.02	0.06±0.02
BW24 (kg)	B	0.03±0.00	0.01±0.00	-	0.09±0.00	0.30±0.04	0.10±0.02	-
BWFE (kg)	A	0.03±0.00	-	-	0.05±0.00	0.47±0.06	-	-
AFE (day)	C	40.49±14.74	-	13.95±7.58	246.37±10.04	0.16±0.06	-	0.06±0.03
EWFE (g)	A	3.03±0.91	-	-	19.40±0.77	0.16±0.05	-	-
EN (egg)	A	24.50±17.52	-	-	161.65±12.00	0.15±0.11	-	-

LHKK, Lueng Hang Kao Kabinburi; σ^2^_a_, direct additive genetic effect; σ^2^_m_, maternal genetic effect; σ^2^_pe_, permanent environmental hen effects.

1) BW1D, body weight at day 1; BW4, BW8, BW12, BW16, BW20, BW24 are body weight at 4, 8, 12, 16, 20 and 24 weeks of age, respectively; BWFE, body weight at first egg; AFE, age at first egg; EWFE, egg weight at first egg; EN, total number of eggs laid from onset of lay to 17 weeks of lay.

2) Model A: **Y** = **Xb**+**Z_1_a**+**e**, Model B: **Y** = **Xb**+**Z_1_a**+**Z_2_m**+**e**, Model C: **Y** = **Xb**+**Z_1_a**+**Z_3_pe**+**e**, Model D: Y = **Xb**+**Z_1_a**+**Z_2_m**+**Z_3_pe**+**e**

**Table 3:** Direct additive genetic (above diagonal) and maternal genetic (below diagonal) correlations (×100) between body weight traits and egg production traits of LHKK chickens

Trait	BW1D	BW4	BW8	BW12	BW16	BW20	BW24	BWFE	AFE	EWFE	EN
BW1D	-	37±10	29±9	27±9	30±10	25±11	35±11	65±10	63±18	71±13	−10±31
BW4	65±14	-	86±3	74±4	60±6	67±6	52±7	55±8	−18±16	46±13	−55±23
BW8	80±15	98±7	-	97±1	90±2	89±3	81±4	77±6	−12±15	53±12	−41±22
BW12	67±16	97±11	97±5	-	98±1	95±1	92±2	78±5	−17±15	37±14	−16±23
BW16	67±13	79±15	98±7	100±2	-	99±1	97±1	81±5	10±17	40±14	−12±26
BW20	71±18	89±17	93±12	100±4	99±2	-	99±1	85±5	2±17	44±14	−21±25
BW24	66±9	100±9	100±5	100±2	100±1	100±0	-	93±4	18±18	49±14	−34±24
BWFE	-	-	-	-	-	-	-	-	72±13	60±12	−24±29
AFE	-	-	-	-	-	-	-	-	-	66±15	−73±36
EWFE	-	-	-	-	-	-	-	-	-	-	−34±35

LHKK, Lueng Hang Kao Kabinburi; BW1D, body weight at day 1; BW4, BW8, BW12, BW16, BW20, BW24 are body weight at 4, 8, 12, 16, 20 and 24 weeks of age, respectively; BWFE, body weight at first egg; AFE, age at first egg; EWFE, egg weight at first egg; EN, total number of eggs laid from onset of lay to 17 weeks of lay.

**Table 4:** Permanent environmental hen effects (above diagonal) correlations (×100) and phenotypic correlations (below diagonal) between body weight and egg production traits of LHKK chickens

Trait	BW1D	BW4	BW8	BW12	BW16	BW20	BW24	BWFE	AFE	EWFE
BW1D	-	31±9	3±10	−13±10	−17±10	−14±9	-	-	−5±16	-
BW4	22±2	-	81±4	58±7	57±7	59±7	-	-	6±19	-
BW8	16±2	68±1	-	90±2	84±3	80±4	-	-	12±18	-
BW12	14±2	56±1	79±1	-	97±1	93±2	-	-	25±18	-
BW16	14±2	47±1	70±1	85±0	-	99±1	-	-	38±16	-
BW20	12±2	47±1	66±1	79±1	90±0	-	-	-	28±17	-
BW24	19±2	39±1	59±1	76±1	85±1	90±0	-	-	-	-
BWFE	14±2	29±3	48±2	58±2	57±2	64±2	63±2	-	-	-
AFE	6±3	−6±3	4±3	1±4	3±4	0.4±4	4±4	36±3	-	-
EWFE	5±2	9±3	12±3	12±3	13±3	15±3	18±3	30±3	38±2	-
EN	−2±4	−7±5	2±6	−9±6	0±6	−6±6	−10±7	−10±8	−9±9	−10±9

LHKK, Lueng Hang Kao Kabinburi; BW1D, body weight at day 1; BW4, BW8, BW12, BW16, BW20, BW24 are body weight at 4, 8, 12, 16, 20 and 24 weeks of age, respectively; BWFE, body weight at first egg; AFE, age at first egg; EWFE, egg weight at first egg; EN, total number of eggs laid from onset of lay to 17 weeks of lay.

**Table 5:** Inbreeding informations, regression coefficient of inbreeding, inbreeding effect on trait mean, and p-value on each trait of LHKK chickens

Trait1)	Total no. of birds	No. of inbred birds (%)	Average inbreeding coefficients2)	SD	Regression coefficient3)±SE	Inbreeding effect on trait mean (% of mean)	p-value
BW1D (g)	11,588	733 (6.33)	0.24	1.45	−0.090±0.02	−0.29	<0.0001
BW4 (g)	11,201	726 (6.48)	0.24	1.45	−0.320±0.24	−0.15	0.26^ns^
BW8 (g)	10,807	721 (6.67)	0.24	1.43	0.630±0.63	0.10	0.48^ns^
BW12 (kg)	9,777	705 (7.21)	0.26	1.46	0.003±0.00	0.27	0.02*
BW16 (kg)	8,948	661 (7.39)	0.26	1.43	0.001±0.00	0.07	0.05^ns^
BW20 (kg)	7,643	580 (7.59)	0.25	1.41	0.002±0.00	0.11	0.39^ns^
BW24 (kg)	6,157	497 (8.07)	0.24	1.26	0.004±0.00	0.19	0.34^ns^

LHKK, Lueng Hang Kao Kabinburi; No. of inbred birds, % of inbred birds from total observations; SD, standard deviation; SE, standard error; ns, non-significant.

1) BW1D, body weight at day 1; BW4, BW8, BW12, BW16, BW20, BW24 are body weight at 4, 8, 12, 16, 20 and 24 weeks of age, respectively.

2) Average for all animals of trait.

3) The regression coefficient of inbreeding level.
