# Supplementary material for: Genetic parameters and inbreeding effects for production traits of Thai native chickens
Source: Asian-Australas J Anim Sci. 2019 Jan 2;32(7):930–8. doi: 10.5713/ajas.18.0690 (PMC6601067; doi:10.5713/ajas.18.0690)
Supplement: Supplementary file 1 [file ajas-18-0690-suppl.pdf]

**SUPPLEMENTAL DATA**

**Supplement Table 1.** Variance components of direct additive genetic effect, maternal genetic effect and permanent environmental hen effects for body weight at day 1 (BW1D), 4 (BW4), 8 (BW8), 12 (BW12), 16 (BW16), 20 (BW20) and 24 (BW24) weeks of age, and body weight at first egg (BWFE) of LHKK chickens using in different models.

| Trait | Model[1] | Variance components | | | | Genetic parameters | | | Log L |
|---|---|---|---|---|---|---|---|---|---|
| | | $\sigma^2_a$ | $\sigma^2_m$ | $\sigma^2_{pe}$ | $\sigma^2_p$ | $h^2_a$ | $h^2_m$ | $h^2_{pe}$ | |
| BW1D (g) | A | 5.98±0.23 | - | - | 10.26±0.17 | 0.58±0.02 | - | - | -17878.51 |
| | B | 1.21±0.23 | 8.35±0.41 | - | 13.77±0.41 | 0.09±0.02 | 0.61±0.01 | - | -16533.65 |
| | C | 1.40±0.24 | - | 5.75±0.28 | 11.27±0.29 | 0.12±0.02 | - | 0.51±0.02 | -16540.11 |
| | **D** | **1.20±0.23** | **2.96±0.51** | **3.13±0.38** | **11.51±0.33** | **0.10±0.02** | **0.26±0.04** | **0.27±0.03** | **-16495.77** |
| BW4 (g) | A | 474.82±32.81 | - | - | 1177.43±20.92 | 0.40±0.02 | - | - | -44206.29 |
| | B | 247.89±33.38 | 160.55±17.91 | - | 1176.82±21.89 | 0.21±0.03 | 0.14±0.01 | - | -44120.98 |
| | C | 249.17±32.27 | - | 127.29±13.11 | 1138.20±19.92 | 0.22±0.03 | - | 0.11±0.01 | -44112.20 |
| | **D** | **232.65±32.32** | **47.98±19.11** | **91.40±17.03** | **1141.78±20.34** | **0.20±0.03** | **0.04±0.02** | **0.08±0.02** | **-44107.08** |
| BW8 (g) | A | 3814.22±243.74 | - | - | 8027.36±151.74 | 0.48±0.02 | - | - | -52729.33 |
| | B | 2679.65±266.64 | 767.97±109.43 | - | 7995.64±157.31 | 0.34±0.03 | 0.10±0.01 | - | -52681.30 |
| | C | 2780.03±261.40 | - | 585.86±78.27 | 7837.23±150.61 | 0.36±0.03 | - | 0.08±0.01 | -52678.94 |
| | **D** | **2645.90±264.98** | **301.01±130.00** | **379.14±104.73** | **7860.71±153.07** | **0.34±0.03** | **0.04±0.02** | **0.05±0.01** | **-52675.10** |
| BW12 (kg) | A | 0.01±0.00 | - | - | 0.02±0.00 | 0.49±0.03 | - | - | -52472.54 |
| | B | 0.01±0.00 | 0.00±0.00 | - | 0.02±0.00 | 0.38±0.03 | 0.08±0.01 | - | -52437.31 |

| | | | | | | | | | |
|---|---|---|---|---|---|---|---|---|---|
| | C | 0.01±0.00 | - | 0.00±0.00 | 0.02±0.00 | 0.38±0.03 | - | 0.07±0.01 | -52433.93 |
| | **D** | **0.01±0.00** | **0.00±0.00** | **0.00±0.00** | **0.02±0.00** | **0.37±0.03** | **0.03±0.02** | **0.05±0.01** | **-52430.75** |
| BW16 (kg) | A | 0.02±0.00 | - | - | 0.04±0.00 | 0.41±0.03 | - | - | 10311.28 |
| | B | 0.01±0.00 | 0.00±0.00 | - | 0.04±0.00 | 0.31±0.03 | 0.08±0.01 | - | 10341.79 |
| | C | 0.01±0.00 | - | 0.00±0.00 | 0.04±0.00 | 0.31±0.03 | - | 0.07±0.01 | 10341.94 |
| | **D** | **0.01±0.00** | **0.00±0.00** | **0.00±0.00** | **0.04±0.00** | **0.30±0.03** | **0.04±0.02** | **0.04±0.01** | **10345.32** |
| BW20 (kg) | A | 0.03±0.00 | - | - | 0.06±0.00 | 0.42±0.03 | - | - | 6988.87 |
| | B | 0.02±0.00 | 0.01±0.00 | - | 0.06±0.00 | 0.31±0.03 | 0.08±0.02 | - | 7010.73 |
| | C | 0.02±0.00 | - | 0.00±0.00 | 0.06±0.00 | 0.30±0.03 | - | 0.07±0.01 | 7014.48 |
| | **D** | **0.02±0.00** | **0.00±0.00** | **0.00±0.00** | **0.06±0.00** | **0.30±0.03** | **0.02±0.02** | **0.06±0.02** | **7015.38** |
| BW24 (kg) | A | 0.04±0.00 | - | - | 0.10±0.00 | 0.46±0.03 | - | - | 4462.07 |
| | **B** | **0.03±0.00** | **0.01±0.00** | **-** | **0.09±0.00** | **0.30±0.04** | **0.10±0.02** | **-** | **4479.15** |
| | C | 0.03±0.00 | - | 0.01±0.00 | 0.09±0.00 | 0.31±0.04 | - | 0.08±0.02 | 4477.75 |
| | D | 0.03±0.00 | 0.01±0.00 | 0.00±0.00 | 0.09±0.00 | 0.29±0.04 | 0.06±0.03 | 0.04±0.02 | 4480.07 |
| BWFE (kg) | **A** | **0.03±0.00** | **-** | **-** | **0.05±0.00** | **0.47±0.06** | **-** | **-** | **-8117.52** |
| | B | 0.02±0.00 | 0.00±0.00 | - | 0.05±0.00 | 0.39±0.08 | 0.05±0.04 | - | -8116.66 |
| | C | 0.02±0.00 | - | 0.00±0.00 | 0.05±0.00 | 0.44±0.07 | - | 0.03±0.03 | -8117.15 |
| | D | 0.02±0.00 | 0.00±0.00 | 0.00±0.00 | 0.05±0.00 | 0.39±0.08 | 0.05±0.06 | 0.00±0.05 | -8116.67 |

[1] Estimates from the best model were bolded.

**Supplement Table 2.** Variance components of direct additive genetic effect, maternal genetic effect and permanent environmental hen effects for age at first egg (AFE) , egg weight at first egg (EWFE) and total number of egg from onset of lay to 17 weeks of lay (EN) of LHKK chickens using in different models.

| Trait | Model[1] | Variance components | | | | Genetic parameters | | | Log L |
|---|---|---|---|---|---|---|---|---|---|
| | | $\sigma^2_d$ | $\sigma^2_m$ | $\sigma^2_{pe}$ | $\sigma^2_p$ | $h^2_d$ | $h^2_m$ | $h^2_{pe}$ | |
| AFE (day) | A | 58.74±14.11 | - | - | 247.97±10.21 | 0.24±0.05 | - | - | -4390.07 |
| | B | 36.91±16.58 | 14.18±8.84 | - | 247.13±10.12 | 0.15±0.07 | 0.06±0.04 | - | -4388.61 |
| | **C** | **40.49±14.74** | **-** | **13.95±7.58** | **246.37±10.04** | **0.16±0.06** | **-** | **0.06±0.03** | **-4387.63** |
| | D | 40.18±17.13 | 0.36±11.34 | 13.74±10.34 | 246.36±10.04 | 0.16±0.07 | 0.00±0.05 | 0.06±0.04 | -4387.63 |
| EWFE (g) | **A** | **3.03±0.91** | **-** | **-** | **19.40±0.77** | **0.16±0.05** | **-** | **-** | **-2697.57** |
| | B | 2.49±1.16 | 0.38±0.59 | - | 19.39±0.77 | 0.13±0.06 | 0.02±0.03 | - | -2697.34 |
| | C | 3.03±1.04 | - | 0.00±0.50 | 19.40±0.77 | 0.16±0.05 | - | 0.00±0.03 | -2697.57 |
| | D | 2.49±1.16 | 0.39±0.83 | 0.00±0.76 | 19.39±0.77 | 0.13±0.06 | 0.02±0.04 | 0.00±0.04 | -2698.00 |
| EN (egg)[2] | **A** | **24.50±17.52** | **-** | **-** | **161.65±12.01** | **0.15±0.11** | **-** | **-** | **-1151.49** |

[1] Estimates from the best model were bolded.

[2] Model with direct additive genetic effect only fitted
